# ‘There are no more secrets’: acceptability of a family-centered model of care for HIV positive children in Eswatini

**DOI:** 10.1186/s12913-020-05810-5

**Published:** 2020-10-15

**Authors:** Philisiwe N. Khumalo, Leila Katirayi, Kim Ashburn, Caspian Chouraya, Lydia Mpango, Nobuhle Mthethwa, Lynne M. Mofenson

**Affiliations:** 1 Elizabeth Glaser Pediatric AIDS Foundation, Mbabane, Eswatini; 2 Elizabeth Glaser Pediatric AIDS Foundation (EGPAF), Washington, DC USA; 3 Eswatini Ministry of Health, Mbabane, Eswatini

**Keywords:** Eswatini, Pediatric HIV, Family-centered care, HIV/AIDS, Children, Qualitative, Stigma

## Abstract

**Background:**

HIV-positive children have lagged adults on retention in HIV care and viral suppression. To address this gap, Eswatini’s Ministry of Health started a pilot family-centered HIV care model (FCCM) targeting HIV-positive children under 20 years old and their families.

**Methods:**

We conducted semi-structured in-depth interviews with 25 caregivers and 17 healthcare workers (HCWs) to assess acceptability of FCCM in four pilot FCCM health facilities in Hhohho region of Eswatini. Thematic analysis with inductive and deductive codes was used to identify salient themes.

**Results:**

Caregivers and HCWs reported FCCM benefits including strengthening the family bond, encouragement for family members to disclose their HIV status and supporting each other in taking antiretroviral drugs. Caregivers reported that they spent fewer days in clinic, experienced shorter waiting times, and received better counseling services in FCCM compared to the standard-of-care services. FCCM implementation challenges included difficulty for families to attend clinic visits together (e.g., due to scheduling conflicts with weekend Teen Support Club meetings and weekday FCCM appointments). Both HCWs and caregivers mentioned difficulty in sharing sensitive health information in the presence of other family members. HCWs also had challenges with supporting caregivers to disclose HIV status to children and managing the larger group during clinic visits.

**Conclusions:**

FCCM for HIV-positive children was acceptable to both caregivers and HCWs, and they supported scaling-up FCCM implementation nationally. However, special considerations should be made to address the challenges experienced by participants in attending clinic visits together as a family in order to achieve the full benefits of FCCM for HIV positive children.

## Background

While there has been progress in prevention of new pediatric infections, in 2018, 160,000 children aged 0–14 years became newly infected with HIV globally, missing the UNAIDS 2018 target to reduce new pediatric infections to under 40,000 [1]. In Eswatini, in 2017, 2.8% of all children aged 0–14 years and 4.1% of those aged 10–14 years were living with HIV [2]. Rates of retention in care and viral load suppression for children are lower than adults in Eswatini; in 2017, 91.4% of adults on antiretroviral treatment (ART) had suppressed viral load compared to 73.9% of children [2]. In 2018, 75% of children were retained in HIV care 6 months after initiation on treatment compared to 78% in adults [3]. Thus, interventions are needed to enhance engagement of children in HIV care and ensure provision and adherence to ART.

Children are uniquely dependent on caregivers to access HIV diagnosis, treatment and care [4]. The family is the main source of support for children in accessing and engaging in HIV care services. Family-centered HIV care models should deliver comprehensive HIV care to all HIV-positive family members in the same clinic visit as opposed to separate pediatric and adult HIV clinics have been proposed [5, 6]. While such models are not new, there has been only limited data on the effect of family-centered care on pediatric outcomes [7, 8]. An evaluation of a family-care model in Uganda found the program did not improve retention but improved adherence to clinic appointment schedule; Qualitative findings from this study suggested participants found family-based-care highly acceptable and felt patient health outcomes benefited from health education and peer support [9]. Challenges have also been identified, including HIV disclosure to children and partners, and difficulties in engaging male partners [5, 10, 11].

In 2016, the Eswatini Ministry of Health (MOH) in collaboration with the Elizabeth Glaser Pediatric AIDS Foundation (EGPAF) initiated a family-centered HIV care model (FCCM) pilot program targeting HIV-positive children 0–19 years old at four health facilities in the Hhohho district of Eswatini to promote better pediatric ART initiation, retention in HIV care and viral load suppression. We conducted a qualitative evaluation of the FCCM program through interviews with caregivers and healthcare workers (HCWs) to understand perspectives on FCCM successes and challenges.

## Methods

### Description of the Eswatini FCCM program

The FCCM provides HIV services comprehensively to families as a unit with the aim of providing more efficient and effective HIV services to promote better ART initiation and retention in families. At health facilities randomized to implement FCCM, HIV-positive children and youth (< 20 years) receiving HIV care and treatment services at FCCM health facilities were enrolled into the program from October 2016 and continues to enroll families. The children were recruited primarily from the HIV care and treatment departments, and in any other units within the health facility such as maternal/child health programs. All HIV-positive children could be enrolled in FCCM, whether they were on ART or not. To enroll in FCCM program, family members had to be willing to disclose their HIV status to other family members, attend clinic visits with the child, and support the child during clinic visits and at home. A family member was defined as someone related to the child either by blood or adoption; or someone residing in the same household who was responsible for the child. Family members living with HIV were invited to receive HIV care at the same facility with the child, if they were already receiving services elsewhere. HIV-negative caregivers were offered non-communicable disease health care services. All family members living with HIV were seen together in the facility and received their HIV care as a family unit at least once a quarter. Health facility based medical records of family members were organized by family and stored together in boxes within the health facilities. ART medications were to be prepared for the family in advance of their visit and families were to be prioritized to be seen (or seen on special clinic “family days”) to decrease waiting time. HCWs were trained in FCCM SOPs, which defined staff purpose, roles and responsibilities, and resources. Table 1 shows the intended FCCM package of services.

**Table 1:** FCCM Package of Services

Service Provided during clinic visits	
• Adherence assessment for all family members (family centered adherence support)	
• Family counselling and assisted disclosure support	
• Patient education in the context of the family to increase patient adherence, treatment and viral load literacy in preparation for long-term adherence and/or a change in ART regimen.	
• Medical history and examination	
• Screening and treating comorbidities/ opportunistic infections	
• Refill ARVs and prophylactic medications (Cotrimoxazole (CTX), Isoniazid Preventive Therapy (IPT), Fluconazole) and adjust ART doses and schedule (same appointments for all individuals in the family unit enrolled in the FCCM)	
• Laboratory monitoring	
• Index testing for family members for ART initiation	
• Referrals for other services (social worker support, nutritional support, cervical cancer screening, family planning etc.)	

### Qualitative study design

One-time semi-structured interviews were conducted with caregivers and HCWs to assess acceptability and feasibility of the FCCM. This qualitative study is a component of a comprehensive evaluation of the effects of the FCCM intervention on viral suppression and retention in care of HIV-positive children called Eswatini FAM-CARE Study [12]. Data collection for the Eswatini FAM-CARE Study was conducted from September 2017 to July 2019. A total 363 unique families (203 in the intervention arm and 160 in the control arm) with of 379 HIV-positive children under the age of 15 years (207 in the intervention arm and 172 in the control arm) from and 28 other family members living with HIV were enrolled in the study. This paper will only focus on the qualitative component.

### Study sites

Interviews took place in the four health facilities implementing the pilot FCCM program in the Hhohho region of Eswatini. The health facilities included one hospital, one health centre and two affiliated clinics, representing higher and lower level of healthcare provision. All facilities were public, government managed facilities. The study sites were selected from all USAID-AIDSFree supported health facilities in Hhohho region. The Hhohho region consists of four clinic “clusters” grouped by a hospital (2 clusters) or health centre (2 clusters) and their affiliated clinics. From the four clusters, two clusters plus the largest affiliated clinic for the cluster were randomly selected to implement the pilot FCCM program and two clusters to continue providing standard-of-care with separate adult and child clinics. Only sites participating in the FCCM were included in the qualitative study.

### Study population

HCWs included General Nurses, Nursing Assistants, Midwives, HIV Testing Services (HTS) Counselors and Expert Clients providing health care services in the FCCM program. FAM-CARE Study Nurses worked with the site nurse-in-charge to identify eligible HCWs (see Table 2 eligibility criteria). In the case that there were more HCWs eligible than the number of interviews selected for that facility, each HCW was assigned a number and selected at random by lottery.

**Table 2:** Eligibility Criteria for Caregivers and HCWs

Caregivers		Healthcare Workers (HCWs)	
Inclusion	Exclusion	Inclusion	Exclusion
• Joined FCCM program at least 12 months prior to data collection • Enrolled in the Eswatini FAM-CARE research study	• Did not participate in FCCM program • Participated in the tool pre-testing	• Provided care in the FCCM for a minimum of 6 months prior to data collection	• Did not provide care in the FCCM program • Participated in the tool pre-testing

Caregivers were selected from a list of caregivers enrolled in both the FCCM program and the Eswatini FAM-CARE Study. At data collection 196 caregivers were both enrolled in the FCCM program and the Eswatini FAM-CARE Study, and 158 had joined the FAM-CARE program at least 12 months prior to data collection for the Eswatini FAM-CARE Study. Caregivers who participated in the study were selected using a computer-aided simple random method. The FAM-CARE Study Nurse contacted the selected caregiver to return on a scheduled day for the interview. Caregivers returning to the facility for the purpose of the interview received transport reimbursement.

The study aimed to interview 15–25 HCWs and 15–25 caregivers. The number of participants to be interviewed was determined following the principle of data saturation. Previous research has found that saturation is reached at 10–12 interviews per homogenous group [12].

### Data collection methods

Semi-structured interview guides developed specifically for the study were used (Additional File 1_IDI Guide for Caregivers and Additional File 2_IDI Guide for Health Care Workers). Topics included experiences enrolling and providing services in the FCCM program, barriers and facilitators within the FCCM program, perceptions about the effect on ART adherence, and strategies to improve the FCCM program. We also collected sociodemographic data for caregivers and healthcare workers. All interviews were conducted between October and December 2018 by local research assistants trained in human subjects’ protections, the study protocol, and qualitative methods. Research assistants were bilingual in English and SiSwati, and familiar with the local context and culture. All interviews with caregivers and HCWs were conducted in private rooms within study sites and conducted either in SiSwati or English depending on the participant’s preference. Participants provided written informed consent before the interview. The duration of the interviews was 30 min to 1 h.

### Data analysis

Interviews were recorded with permission from the participants. The audio-recordings of the interviews were simultaneously transcribed and translated to English by the research assistants who conducted the interviews. The study team reviewed the transcripts and created a codebook using both an inductive and deductive approach, using pre-identified themes and being open to new themes emerging in the data. The transcripts were uploaded and coded in the qualitative software program MAXqda V18. To develop a standardized approach for coding among the team, first, a few transcripts were collectively coded and discussed by the team. Second, a small subset of transcripts coded individually was reviewed by the study coordinator and study investigators, and the feedback was discussed collectively. Questionable segments of coded text were resolved among the coders or by one of the co-investigators leading this qualitative evaluation. After coding was complete, data reduction and summary tables were generated. Data were summarized through descriptive, text-based summaries and tables by study investigators. Transcripts were carefully read by investigators to identify recurrent patterns and themes and to draw conclusions from issues connected to study questions. Results were analyzed by group (caregivers and HCWs) and summarized into overall findings.

### IRB approvals

The FAM-CARE study was reviewed and approved by Population Council Institutional Review Board (IRB) and the National Health Research Review Board in Eswatini.

## Results

The results of the qualitative analysis are organized by themes including participants’ views of the benefits of FCCM, the challenges experienced in engaging in the program, participants’ opinions on whether or not FCCM should be scaled up nationally, and participants’ recommendations on how to improve the program.

### Demographic characteristics of caregivers and health care workers

Interviews were conducted with 25 caregivers and 17 healthcare workers (HCWs). The mean age for caregivers was 37.8 years and ranged from 24 to 63 years (Table 3). Only 8 % (*n* = 2) of the caregivers were male. Fifty-six percent (*n* = 14) of the caregivers were married either in a non-polygamous marriage (40%, *n* = 10) or in a polygamous marriage (16%, *n* = 4). Thirty-two percent (*n* = 8) were never married and 12% (*n* = 3) were cohabiting. A high proportion (76%, 19) of the caregivers were unemployed. Sixty-four percent (*n* = 16) of the caregivers were biologic parents of the children enrolled in FCCM; 76% 9 *n* = 19) came from families in which only the child and the caregiver were HIV-positive and 24% (*n* = 6) came from families with three or more HIV-positive family members. All caregivers interviewed reported that they and their children were receiving ART.

**Table 3:** Demographic Characteristics of Caregivers and Health Care Workers

**Characteristics of caregivers (*N*** **= 25)**	
	**n (%)**
Age (years) Median = 38 (Range: 24–63)	
Sex	
Female	23 (92)
Male	2 (8)
Highest level of school attended	
Primary and lower	12 (48)
Secondary and higher	13 (52)
Marital status	
Never married	8 (32)
Married, non-polygamous	10 (40)
Married, polygamous marriage	4 (16)
Cohabiting	3 (12)
Employment status	
Unemployed	19 (76)
Employed	6 (24)
Relationship to child	
Biological parent	16 (64)
Family members enrolled in FCCM	
Two (caregiver and child)	19 (76)
Three or more family members	6 (24)
Child currently on ART	
Yes	25 (100)
Caregiver currently on ART	
Yes	25 (100)
**Characteristics of Health Care Workers (HCWs) (*N*** **= 17)**	
	**n (%)**
Sex	
Female	14 (82)
Male	3 (18)
Highest level of school attended	
Secondary	2 (12)
High School	1 (6)
Tertiary	14 (81)
Current role in health facility	
Senior Nurse	1 (6)
Midwife	6 (35)
General Nurse	1 (6)
Nursing Assistant	1 (6)
Expert Client	6 (35)
Nursing Sister	2 (12)

Most HCWs were female (82%, *n* = 14). An equal proportion of HCWs were midwives and Expert Clients (35%, *n* = 6). Other HCWs included nursing sister (12%, *n* = 2), senior nurse (6%, *n* = 1), general nurse (6%, *n* = 1) and nursing assistant (6%, *n* = 1).

### FCCM benefits

Both caregivers and HCWs reported they found FCCM highly beneficial in several ways, including strengthening the family bond, improving HIV disclosure, and improving quality of health services provision. Quotes illustrating the FCCM benefits thematic areas are presented in Table 4.

**Table 4:** Illustrative quotes by caregivers and health workers on FCCM benefits

FCCM benefits thematic area	Illustrative quote
Strengthening of family bond	*“…We call each other and remind ourselves that it is 7 and we drink our pills…the child also drinks his pills…We put the pills together on the table one for the child and others for us adults so when we are about to drink we give him his pills.”* (Female caregiver, age 34, of boy age 8)
	*“…The child is able to see that the mother is also on medication and the child will be encouraged because the medication is* [kept] *in the same place.”* (Healthcare worker, health clinic)
	*“Another thing is that there is no more hiding from each other to take pills on time. Even my child is always reminding us that it is now time to take pills, so we do things together. No one to say, eish…now it is time for medication, the child also knows the dates. Even in January the child will come again for the next visit. The family is always free to do things together. It is like a game to us now because we are happy.”* (Female caregiver, age 29, of boy age 8)
Increased HIV status disclosure	*“I had some difficulties to talk with my family, I was hiding from my own husband about my son who is also HIV-positive, so I didn’t have a way to disclose about my son’s HIV status to my husband. My worry was that he is not the biologic father of this child. But when I came to this program, they explained a lot of things to me pertaining to health and that is when I came to know some new things, I took a sound decision that we need to be one as a family, myself and my husband and the child) and then we began to live a healthy and happy life.”* (Female caregiver, age 29, of boy age 8)
	*“The children, with those who are not* [participating in] *FCCM you find that there is a problem of disclosure yet under FCCM the issues can easily be addressed. The viral load is suppressed as compared to the patients who did not join.”* (Healthcare worker, hospital)
Better care in FCCM compared with standard of care	*“And what I like with FCCM program is that, if there is one member who doesn’t drink the medication or doesn’t adhere, the HCWs are able to correct our mistakes, motivate us and encourage us together as a family.”* (Male caregiver, age 40, of girl age 13)
	*“…in case you have forgotten to come to the clinic, they then call you to find out if you still remember about your next visit date. What I can say is that if maybe it wasn’t for FCCM, no one would remind to come for the medication and then you can default in a way or stop coming for the services.”* (Female caregiver, age 28, of boy age 10)
	*“If I may give an example, once we arrive, the nurse will quickly recognize us and see the young one, she will attend to her [the HCW] ‘Auntie’ or ‘Umshana’ (my child). There is that bond between the nurse and the child as they get to know one another or if they meet every time we visit the clinic.”* (Female caregiver, age 28, of girl age 6)
	*“Under the FCCM you address the identified issues one by one and you come up with a long-lasting solution after you have discussed with them because you will have the caregiver around for* [the clinic visits with the child] *she will tell you the challenges that she might have.”* (Healthcare worker, hospital)

#### Strengthening of family bond

Participants perceived that FCCM improved unity among family members and strengthened the family bond. Receiving health services, counselling and instructions from HCWs together as a family helped family members to be aware of and to understand the needs of each other. This put families in a better position to support one another. Family members reminded each other when one forgot clinic appointments, clarified for each other instructions from HCWs and encouraged each other to stay adherent to ART. HCWs said that children liked the FCCM because they got to know that their parents were also taking ART, and that they were not alone in this life circumstance. The children valued taking HIV medication together with adult family members as it created a sense of the family ‘is in it together’. Because families discussed HIV treatment and related issues together, they stopped feeling that there are secrets kept within the family.

#### Increased HIV status disclosure

Participating in the FCCM required discussing HIV and ART as a family unit, which encouraged disclosure. Participants reported that with reduced stigma and discrimination between family members, they felt that family members were able to talk more openly about living with HIV and taking ART, and to support one another to remember to take their medication. HCWs felt that the increased disclosure resulted in better health outcomes. HCWs also became more aware about the need for strengthened support to caregivers to disclose HIV status to children.

#### Better care in FCCM compared with standard of care

Caregivers felt that better care was provided in the FCCM compared to standard-of-care. In FCCM caregivers reported experiencing good counselling, and they found that HCWs were always willing to respond to questions. Caregivers liked that in FCCM HCWs called them on the phone to remind them of clinic appointments, prioritized them for services provision during clinic visits, were flexible when setting up clinic appointments compared to standard of care, and kept their medical records together organized in boxes. The family preferred the box system because they did not have to wait too long for the HCWs to locate their medical records.

In FCCM, families were often provided health services by the same HCW at each visit. Participants felt that being provided health services by the same HCW built rapport with the HCWs and strengthened the relationship families had with HCWs. This made families feel like they had their own doctor and nurse which they could count on. Caregivers felt that FCCM is the preferred model if they wanted to stay healthy.

HCWs reported that FCCM helped them to get a more comprehensive history of the patients, and each family member’s performance on HIV treatment and challenges related to taking medication. This helped them to identify long lasting solutions for patients.

### FCCM challenges

There were several challenges faced by HCWs and caregivers with FCCM, including attending clinic visits as a family, participation of male family members, disclosure and HCW service provision challenges. Quotes illustrating the FCCM benefits thematic areas are presented in Table 5.

**Table 5:** Illustrative quotes by caregivers and health workers on FCCM challenges

FCCM challenges thematic area	Illustrative quote
Attending clinic visits as a family	*“It becomes impossible, of which some families they have never came together as a family. A child comes in alone to the teen club, parents come in by themselves mid-week to the facility, of which we then don’t meet goals of FCCM. FCCM says we need to see them at least twice a year together but we couldn’t do all that.”* (Healthcare worker, health centre)
	*“The other thing is this FCCM is new to us. Children are used to coming to the facility on their own. The challenges we encountered when we first started the program were that kids get the medication according to their weights, refills for 1 month depending on how the kid has taken the medication and then increase to 2 months. That made the caregivers not to understand why they were now given 1 month instead of 3 months. Since the FCCM was introduced, every caregiver is entitled to a month [of medication]. We try to explain all that to them as we more concerned about the kids’ health and to know what is going on; is there any improvement, we try to monitor everything on the child as she is the brighter future of the nation.* (Healthcare worker, hospital)
	*“The challenges that we always come across are that at times in the family there will be a certain individual who will always send others to pick his/her medication, yet we need to do her/his Labs (tests) if they are already due. As much as you need to see that particular patient, it then becomes a bit difficult for us as health care workers and as for FCCM it was meant for family people to be seen together or schedule the appointments at the same time.”* (Healthcare worker, health centre)
Participation of male family members	*“I think he knows it, but he does not want to take ART because he ran away even in this facility because they wanted to test him because they know my status and the status of the child.” (*Female caregiver, age 32, of girl age 10)
	*“Males or some males don’t want to come, they don’t want to come with their children or maybe as a family they want to come alone. Others will tell you they work very far in South Africa, so they come on the schedule date, they want their own date so that they come alone. They don’t come as a family.”* (Healthcare worker, health clinic)
Health care work service provision challenges	*“At times you find it difficult as there are some instances whereby you cannot just ask questions of a caregiver in front of the kid if you will need to fill in the chronic care file, asking a man or woman about things like STI [sexually transmitted infection] screening and family planning model, it is not easy to ask such questions in front of kids.”* (Healthcare worker, hospital)
	*“I think FCCM just needed nurses that were specifically for the program not doing FCCM, refills or everything else. So, we really needed a nurse specifically for FCCM. I just feel like FCCM needed attention, so we really could not focus on FCCM because we also have other things to do in the facility and you find that the people that are not on FCCM they are not taken care of like those who are in the program.”* (Healthcare worker, hospital)

#### Attending clinic visits as a family

Both caregivers and HCWS said there were scheduling conflicts with clinic visits for children attending school and psychosocial support groups (Teen Clubs) for adolescents on Saturdays. This made it difficult for families to attend clinic visit together as a family. Additional costs were also incurred when families had to cover transport cost for several people at once so that all family members could visit the health facility together on the same day, and when caregivers had to visit the health facilities every month with the child. Some HCWs were not comfortable to provide multiple-month refills ART drugs for children, and the HCWs preferred seeing children every month so that they could monitor the weight of children to adjust ARVs dosage accordingly. At some facilities, HCWs said that some families did not attend clinic visits together as a family unit, instead they would send one person to pick up medication, similar to community adherence groups (CAGs). This practice went against the principle of a FCCM.

#### Participation of male family members

Caregivers also had difficulty with encouraging men in their families to participate in FCCM because men did not want to get an HIV test or disclose their HIV status.

#### Health care work service provision challenges

For HCWs, it was a challenge to ask sensitive questions, such as asking adolescents about their sexual activity in the presence of their caregiver or asking caregivers about reproductive health and family planning in front of their children. HCWs also had challenges with managing larger groups of family members at the same time, particularly if family members did not get along. When there were larger groups and staff members were seeing multiple clients at one time, the clinic visits took longer, and some facilities did not have enough staff to cover the visits.

### Perspectives and recommendations on national scale-up of FCCM

The majority of caregivers and HCWs recommended national implementation of FCCM. They felt that FCCM improves HIV disclosure among the family, reduces stigma and discrimination within the family and aids in adherence to HIV treatment and clinic appointments. HCWs suggested adding more staff and also offering better and more frequent supplemental training for HCWs for supporting disclosure and how to manage larger groups in providing family centered clinic visits. HCWs also recommended adding a social worker to the FCCM staff to address psychosocial needs of families.

## Discussion

Our study provides some of the first published qualitative findings on acceptability of FCCM for HIV-positive children. We found that FCCM was acceptable to both caregivers and HCWs Both HCWs and caregivers strongly valued the FCCM program and felt that it improved patient’s well-being, both physically and psycho-socially.

Caregivers and HCWs felt that FCCM strengthened relationships and health information sharing for families, and between family members and HCWs. This finding is similar to a finding in an exploratory, qualitative study among adult patients with chronic diseases in an out-patient department in Nigeria where participants perceived FCCM to foster ‘family ties and to build relationships which includes the doctor as an integral part of the family’ [13]. Improved family relationships and family-HCW interactions could improve adherence to HIV treatment, retention in HIV care and clinical outcomes for HIV-positive children [7, 8, 14]. Additionally, improved partnership between children, caregivers and their HCWs can promote transparency, truthfulness, and promote HIV status disclosure [15].

Participants reported that FCCM encouraged disclosure of HIV status among family members. A study in Thailand reported a similar result, where a FCCM program improved readiness and willingness of parents to disclose their HIV status to their children, and improved HIV disclosure among families [16]. Disclosure of HIV status among family members can have important benefits for treatment, care and support for both children and adults living with HIV, improving communication between family members and providing social support, which may improve adherence to HIV treatment and improve health outcomes [16–18]. In the FCCM program, children learned that their caregivers were also taking HIV treatment. This may encourage and motivate children to stay on HIV treatment.

HCWs mentioned inadequacies on their part to assist parents to disclose HIV status to children. HCWs reported the process of HIV status disclosure for children was complex, confusion about their role and responsibility in the process of disclosing to the HIV-positive child, and unclear guidelines and lack of training in pediatric HIV disclosure. Similar findings have been obtained in other settings [19, 20]. This is compounded by hesitation from parents to disclose HIV status to children due to lack of disclosure skills, concerns about causing psychological distress to children, guilt for having infected their children, fear of diminished capacity of children to keep secrets which may lead to disclosure to other people leading to stigma and discrimination, and diminished capacity of children to understand the whole concept of HIV and AIDS due to young age [19, 21–25]. Training and mentorship for HCWs and provision of instructional materials such as job aids and a curriculum on how to assist parents to disclose HIV status to their children can assist to improve confidence in HCWs to assist HIV disclosure by caregivers to children.

Both caregivers and HCWs experienced some difficulties with FCCM. HCWs had concerns as families were not visiting health facilities together as a unit as required by FCCM principles [15, 26, 27] and by how the FCCM pilot program was designed. HCWs struggled synchronizing clinic visits appointments dates for families mainly due to schedule conflicts for school-going HIV-positive children and teenagers participating in weekend Teen Clubs for psychosocial support. HCWs were also not comfortable with aligning clinic visits for children to those of adults receiving multiple-month ART refills, wanting to see children on a monthly basis to monitor child development and adjust dosage accordingly despite the 2010 Swaziland Integrated HIV Management Guidelines [28] which recommends that children are offered multiple monthly refills if they are stable. In other cases, some families made it a habit to send one family member to pick up medication for all family members defeating the principle of a family-centered care. In general, there was a lack of program fidelity, only about 40% of all 465 families enrolled in the FCCM program at the pilot sites actually attended at least one clinic visit together as a family, and only about 26% of families attended clinic visits together as a family more frequently at four times a year.

Other challenges noted included potential additional costs for clinic visits with multiple family members; lack of participation of men in the families; staff difficulty managing larger groups of family members at the same time and discussing sensitive health information in presence of other family members. Similar challenges to family-centered models of care have been reported in other studies [8, 29].

### Study limitations

We did not interview children and adolescents to gather their experiences and opinions about FCCM and fewer male caregivers were interviewed for the study due to fewer male caregivers being involved in the overall program. This limited the understanding of how the FCCM was experienced and perceived by these other users. In addition, the study did not attempt to contact any FCCM participants that may have dropped out of the program. It is possible that these views are not fully represented in this study. Because available HCWs were recruited on the day of data collection, no medical doctors were interviewed for the study, although they provided health services in FCCM. Low program fidelity may have resulted in many participants not experiencing the full benefits of the FCCM program.

## Conclusion

Pediatric HIV presents complex challenges that requires taking social context of the child into consideration due to children’s dependency on their caregivers. The FCCM provides an opportunity to improve the child and family’s health outcomes, in addition to strengthening the bonds of the family to increase their own social support to each other. FCCM was well-liked by both caregivers and HCW, but special considerations should be made to address the challenges experienced by participants in attending clinic visits together as a family. A need for HCW training in promotion of disclosure and how to optimize psychosocial support was noted and scheduling barriers for children related to school and Teen Clubs need to be addressed.

## Supplementary information

**Additional file 1.** IDI Guide for Caregivers.

**Additional file 2.** IDI Guide for Health Care Workers.

## Data Availability

The data generated and analyzed during the study are available from the corresponding author on reasonable request.

## Abbreviations

AIDS: Acquired Immunodeficiency Syndrome
ARVs: Antiretroviral Drugs
ART: Antiretroviral Treatment
CTX: Cotrimoxazole
EGPAF: Elizabeth Glaser Pediatric AIDS Foundation
FCCM: Family-Centered HIV Care Model
HCWs: Healthcare Worker
HIV: Human Immunodeficiency Virus
IPT: Isoniazid Preventive Therapy
MOH: Ministry of Health
SOAR: Supporting Operational AIDS Research
STI: Sexually Transmitted Infection
UNAIDS: United Nations Programme on HIV/AIDS
USAID: United States Agency for International Development
