# Supplementary material for: ‘There are no more secrets’: acceptability of a family-centered model of care for HIV positive children in Eswatini
Source: BMC Health Serv Res. 2020 Oct 15;20:951. doi: 10.1186/s12913-020-05810-5 (PMC7559472; doi:10.1186/s12913-020-05810-5)
Supplement: Supplementary file 1 — Additional file 1. IDI Guide for Caregivers. [file 12913_2020_5810_MOESM1_ESM.docx]

**In-depth Interview Guide: Caregivers**

***Read to the participant:*** Now are going to move to more of a discussion. We’ll talk about how you feel about the family-centered care model. This is referring to the new model where we whole family come to the facility together to receive HIV care and services instead of the woman coming separately, the child coming for separate appointments and other family members coming separately. This is the new model, called the family-centered care model. Are you familiar with this model?

*Confirm that that participant understands what the family-centered model is before proceeding to the questions below. This is very important.*

**Feedback on the family-centered care model**

1. What were your initial thoughts when you were told about the family-centered model?

*Let the participant respond and then use the following probes:*

- Were you excited or hesitant to participate? Please explain why.
- Did you have any initial concerns? If yes, please tell me about your initial concerns.

1. How was your experience enrolling in the program?

*Let the participant respond and then use the following probes:*

- Did you face any challenges enrolling in the program? If yes, please tell me about the challenges.

1. Once you were enrolled in the program, how did you feel about the care that you, your child and other HIV-positive family members received in the program?

*Let the participant respond and then use the following probes:*

- How well did you feel that the needs of the different family members were met?
- How did you feel about the services provided in the model?
  - Were there additional services you wished to receive?

1. After your initial enrollment in the family care program, how did the quality of services that you, your child and other HIV-positive family members received change? Please think back over the last year and a half and describe any differences to me.

*Let the participant respond and then use the following probes:*

- Do you feel that you continued to receive enough information about you and your child’s health and medications?
- How easy was it to book appointments as a family and continue to be seen as a family?

1. How do you think participating in this model has affected your decision for you, your child and other HIV-positive family members to continue to take antiretroviral therapy?

*Let the participant respond and then use the following probes:*

- Have you noticed a difference your child and other HIV-positive family members ability to take antiretroviral therapy since enrolling in the family program? Please describe the differences.

1. How do you think participating in this model has affected your decision for you, your child and your family to continue receive care at the facility?

*Let the participant respond and then use the following probes:*

- Has it been more or less convenient to receive HIV care and treatment services as a family? Please explain why.

1. Please tell me about any challenges you have experience being in the family care program.

*Let the participant respond and then use the following probes:*

- Challenges with disclosure
- Challenges with attending the facility as a family
- Challenges with picking up antiretroviral therapy for family members
- Challenges with service provision
- Challenges with continuing to receive HIV services as a family
- Negative attitudes from HCWs at the facility
- Negative attitude from other ART clients not in the FARM-CARE program
- Any other challenges?

1. How do you feel about the health care workers providing services in the family care model?

*Let the participant respond and then use the following probes:*

- How well do you feel that the HCW was able to address the needs of all family members?
- Do you feel that you received enough information to understand your child’s health status and the health status of other HIV-positive family members? Why or why not?
- Do you feel that you received enough information about ART for your child’s health and the health of other HIV-positive family members? If not, what additional information would you have liked to receive?
- How friendly was the HCW towards you and your child when receiving services?

1. How did you feel about the space in which you received services in the family care model?

*Let the participant respond and then use the following probes:*

- Was the space appropriate for children and adults? Why or why not?
- How well did you feel that your privacy was protected?
- How comfortable was the space?

1. How can we make the family care program better?

*Let the participant respond and then use the following probes:*

- Suggestions around the timing of the appointments (are current day/times okay?), suggestions on HCW providing services, additional services needed, additional information needed, frequency of appointments, etc.

1. Would you recommend the family care program to other families? Why or why not?

This brings us to the end of our interview. Do you have any questions? Thank you for your time.
