# Supplementary material for: ‘There are no more secrets’: acceptability of a family-centered model of care for HIV positive children in Eswatini
Source: BMC Health Serv Res. 2020 Oct 15;20:951. doi: 10.1186/s12913-020-05810-5 (PMC7559472; doi:10.1186/s12913-020-05810-5)
Supplement: Supplementary file 2 — Additional file 2. IDI Guide for Health Care Workers. [file 12913_2020_5810_MOESM2_ESM.docx]

**In-depth Interview Guide: Health Care Workers**

All directions for the RA are provided in *italics.* Please do not read anything in italics to the participants.

***Read to the participant:*** Now are going to move to more of a discussion. We’ll talk about how you feel about the family-centered care model. Are you familiar with this phrase? This is referring to the new model where we whole family come to the facility together to receive HIV care and services instead of the woman coming separately, the child coming for separate appointments and other family members coming separately. This is the new model, called the family-centered care model. Are you familiar with this model?

**Feedback on the Family Centered Care Model**

Questions about Service Provision

1. When you initially learned about the family-centered care model, what were your initial thoughts?

*Let the participant respond and then use the following probes:*

- Did you think it was a good idea or a bad idea? Why did you think this?
- What potential challenges did you anticipate?

1. How did you feel about the training you received to provide family centered care?

*Let the participant respond and then use the following probes:*

- How well did you feel prepared to provide care to the different family members?
- Were you comfortable providing services to the children?
- What components of the family centered care would you have liked to receive more training on?

1. As a HCW providing services in the family centered care model, what challenges did you experience

*Let the participant respond and then use the following probes:*

- Challenges with enrolling families into family centered care, providing services to the families, stock-out of supplies needed in the family centered care model, challenges with space, overlap of roles and responsibilities at the facility, loss to follow up of clients, comfortability with working in the family centered care model, etc.

1. What would make it easier for you to provide family centered care?

*Ask participants to speak freely and provide any suggestions. Remind them that we are here to learn from them.*

Questions about Clients

*Tell the participants that now we are going to talk about the clients that attend the family centered care model.*

1. When you initially inform clients about the family centered care model and invite them to join, what are their initial reactions?

*Let the participant respond and then use the following probes:*

- Are they excited or concerned about joining?
- What are some of the reasons that they may be excited to join?
- What are some of the reasons that they may be concerned to join?
- What questions do they have about the program?

1. Once clients are enrolled in the family centered care model, what are their general perceptions towards the family centered care program?

*Let the participant respond and then use the following probes:*

- What do they like about the program?
- What do they not like about the program?

1. Can you tell me more about challenges that clients have with participating in the family centered care model?

*Let the participant respond and then use the following probes:*

- Challenges with the scheduling of the appointments, disclosing their status, returning for the scheduled appointments, picking up ART, etc.

1. How do you think the family centered care model has affected clients’ motivation to stay on antiretroviral therapy?

*Let the participant respond and then use the following probes:*

- Please think back to clients who you cared for who did not participate in the family centered care program, when comparing clients in the family centered care program to those who have not participated in the program, how do you think the family centered care program affects clients’ motivation to stay on antiretroviral therapy?
- Can you tell me any examples?
- Why do you think there is this effect?

1. How do you think the family centered care program has affected clients’ interest in continuing to receive care at the facility?

*Let the participant respond and then use the following probes:*

- Please think back to clients who you cared for who did not participate in the family centered care program, when comparing clients in the family centered care program to those who have not participated in the program, how do you think the family centered care program affects clients’ interest in continuing to receive care at the facility?
- Can you tell me any examples?
- Why do you think there is this effect?

1. What recommendations do you have to improve the family centered care program?

*Let the participant respond and then use the following probes:*

- Structure of program, services provided, schedule of visits, requirements for participation in the program, advertising of the program, etc.

1. After your experience providing care in the family centered care model, do you think that this model should be rolled out across Swaziland? Why or why not?

This is the end of our interview. Do you have any questions? Thank you for your time.
